# *VPS16*-Associated Dystonia: A Cohort-Based Clinical, Imaging and Genetic Profile

**DOI:** 10.5334/tohm.1030

**Published:** 2025-07-07

**Authors:** Rohan R. Mahale, Hansashree Padmanabha

**Affiliations:** 1 Department of Neurology, National Institute of Mental Health and Neurosciences, Bengaluru, India

**Keywords:** dystonia, VPS16, HOPS complex

## Abstract

**Background:**

Monoallelic variants in *VPS16* are associated with early-onset dystonia (*VPS16*-associated dystonia) with a frequency of less than 4%.

**Objective:**

Description of the clinical, imaging, and genetic profile of *VPS16*-associated dystonia and comparison of the findings of the Indian cohort with that of the Chinese and European cohorts.

**Methods:**

Report of a single patient with *VPS16*-associated dystonia and review of reported cases of genetically confirmed DYT-*VPS16* since 2016 from Indian, Chinese and European cohorts.

**Results:**

There were a total of 3 cases from India, 10 cases from China, and 34 cases from Europe. The median age at onset was similar in all cohorts. The median duration of disease was 26 years in the European cohort but a shorter duration was noted in the Indian and Chinese cohorts (13–14 years). Dystonia was the common symptom observed in all cohorts with associated myoclonus, intellectual disability, and psychiatric symptoms commonly reported from European cohort. The onset of dystonia was primarily noticed in the limb/cervical region in all cohorts. Partial to good response to globus pallidus interna deep brain stimulation (DBS) was reported from the European cohort. Brain magnetic resonance imaging was usually normal in all the cohorts. Frameshift and stop-gain variants were common in the European and Indian cohorts, whereas missense variants were common in the Chinese group. Segmental dystonia was common in frameshift variants (70%) and generalized dystonia in stop-gain variants.

**Conclusion:**

Our study showed geographically diverse differences in the phenotypic presentation of *VPS16*-associated dystonia as myoclonus, intellectual disability, and psychiatric symptoms were more common in the European cohort. Segmental dystonia was commonly observed in the frameshift variants and generalized dystonia in stop-gain variants.

**Highlights:**

*VPS16*-associated dystonia is a new genetically mediated dystonic syndrome. The clinical presentation is heterogeneous in terms of intrafamilial and interfamilial variability of age at onset and severity of dystonia. We aimed to study the ethnic phenotypic and genotypic differences of *VPS16*-associated dystonia. Our study showed geographically-diverse differences in the phenotypic presentation as myoclonus, intellectual disability, and psychiatric symptoms were more common in the European cohort. Patients with frameshift variants had segmental dystonia, whereas those with stop-gain variants had generalised dystonia.

## Introduction

*VPS16*-associated dystonia (OMIM ID: 619291) is associated with monoallelic variants in *VPS16* gene. The frequency of *VPS16*-associated dystonia is estimated to be between 0.9 and 4% of genetic dystonias. The usual clinical manifestation begins in childhood/young adulthood, starting with focal dystonia, involving the neck or limbs, and gradually progressing to generalized dystonia. The other movement disorders reported include myoclonus, choreoathetosis, and freezing of gait. In addition, peripheral neuropathy, intellectual disability, or neuropsychiatric manifestations including mood disorders and impulsivity are other neurological symptoms reported [2]. The manifestations are diverse due to intra and inter-familial variability in terms of age at onset and severity of dystonia. Magnetic resonance imaging (MRI) can be either completely normal or abnormal, with evidence of iron deposition in the globus pallidus and dentate with cerebral atrophy. VPS16-associated dystonia can be inherited either as an autosomal dominant pattern with incomplete penetrance or recessive inheritance. *VPS-16* associated dystonia have primarily been reported from Europe and China, but the reports from the Indian continent are lacking. The study aims to provide a description of the clinical, imaging, and genetic profiles of patients with *VPS16*-associated dystonia and to compare the findings of Indian cohort with that of Chinese and European cohorts.

## Methods

### Patients and methods

This was a retrospective cross-sectional, descriptive analysis of patients with *VPS16*-associated dystonia conducted at a quaternary care hospital in India. Hospital records of patients with genetic confirmation of *VPS16*-associated dystonia between 2018 and 2023 were searched and included. The following clinical data were extracted which included age at clinical presentation, age at onset of symptoms (age at which the first neurological sign had been noted) duration of the disease (onset of first symptom to the time of presentation), sex, description of symptoms, birth history, consanguinity, family history and clinical examination findings. A detailed description of dystonia regarding the initial site of onset—cranial/oro-mandibular, cervical, or brachial/crural; progression to segmental/generalized dystonia; and time from onset to generalization and ambulatory status was collected. Brain MRI was analyzed to look for abnormalities that include the presence of atrophy and mineralisation. Details were gathered about the *VPS16* variants if it was missense, frameshift, stop-gain, or splice-site loss, and the zygosity. The study was conducted with the approval of the ethics committee (No: NIMH/DO/DEAN (Basic Science)/2020–21). Informed written consent was obtained from the patients for publication. The study was conducted in accordance with the Declaration of Helsinki which was revised in 2013.

#### Genetic analysis

Genomic DNA extraction was carried out using QIAamp DNA Blood Mini Kit (Qiagen Germany, #51104) on the blood samples of the recruited patients. For the identification of variants, we employed the Genome Analysis Toolkit (GATK) framework (Broad Institute, Cambridge, MA, USA). The variants were annotated utilizing the online platform, ANNOVAR (http://www.openbioinformatics.org/annovar/).34791404]. The common variants with a minor allele frequency (MAF) >0.01 were not considered for the analysis. A comparison was made with the Exome Aggregation Consortium (ExAC), 1000 Genome project, and gnomAD databases (https://gnomad.broadinstitute.org/). The individual sequence variants were interpreted using the PolyPhen-2, Sorting Intolerant from Tolerant (SIFT) webserver, and the MutationTaster. The variants were classified as per the American College of Medical Genetics and Genomics (ACMG) standards and guidelines into benign, likely pathogenic or pathogenic variants. The variants were checked for their novelty by analysis in the mutation databases (ClinVar).

#### Review of literature

We conducted a comprehensive search across the publicly accessible medical database of PubMed, using specific medical subject headings (MeSH): “VPS16,” “VPS16-associated dystonia,” and “HOPS-associated neurological disorders” to identify relevant studies. Initially, studies underwent a rigorous title and abstract screening process. Subsequently, we included studies focusing on the Chinese and European populations for further analysis. We incorporated data from our current study along with previously published cases from India, and compared these findings with that of the Chinese and the European cohort that was last updated in 2023. The data extraction process involved the retrieval of critical information, including demographic parameters, clinical phenotype descriptions, and variant details, to facilitate a comprehensive evaluation of the existing literature on this topic. Studies not presented in English or lacking patient details were excluded from consideration.

#### Statistical analysis

Summary statistics were used to describe the clinical characteristics. The continuous variables were expressed as mean/median and categorical variables as frequency/percentage.

## Results

We had 1 patient with *VPS16*-associated dystonia available for assessment in our cohort. Two cases of *VPS16*-associated dystonia have been previously published by our institute. The findings of the Indian cohort of 3 patients was compared with that of the Chinese and the European groups. One patient included in this study was a male of 34 years with onset at 30 years of age. The duration of symptoms was 4 years.

### Clinical and imaging findings

The patient was born out of non-consanguineous parentage with a normal perinatal history. The initial symptoms were dystonia, which started in the right upper limb, progressed to involve the left upper limb, and occurred during rest with worsening on action. There was no diurnal variation in the dystonic symptom. There were no associated psychiatric symptoms, seizures, or myoclonus. There were no clinically apparent neuropsychological deficits. There was no positive family history. Formal neuropsychological testing was not done. Ophthalmological and auditory evaluations were normal. Both patients had gaze-evoked jerky horizontal nystagmus. The speech was normal. The patient had bi-brachial dystonia in the form of internal rotation of the arm with pronated forearms and flexion of fingers. There were no pyramidal signs and gait was normal. Neuroimaging was normal.

### Genetic findings

Whole-exome sequencing showed a heterozygous 2 base pair deletion in exon 21 of the *VPS16* gene (chr20:g.2865313_2865314del; Depth: 140x) that resulted in a frameshift and premature truncation of the protein 44 amino acids downstream to the codon 724 (p.Lys724GlufsTer44; ENST00000380445.8) (Table 2). The variant has not been reported in the 1000 genomes, gnomAD (v3.1), gnomAD (v2.1) and topmed. The in-silico prediction of the variant was known to be damaging by MutationTaster2. This variant was classified as likely pathogenic (ACMG Criteria: PVS1, PM2).

### Other cases from Indian cohort (n = 2) (Table 1) [3,4]

**Table 1:** Comparison of clinical features, imaging and genetic findings among the cohorts.


	INDIAN COHORT (n = 3)	CHINESE COHORT (n = 10)	EUROPEAN COHORT (n = 34)

Age at presentation (years) (range)^a^	31.8 ± 2.9 (29–34)	32.8 ± 18.1 (9–65)	40.9 ± 17.6 (7–76)

Age at onset (years) (range)^a^	16.5 ± 8.5 (10–30)	16.9 ± 12.4 (8–60)	14.9 ± 11.4 (3–52)

Duration (years) (range)^a^	11.3 ± 8.9 (4–26)	12.4 ± 7.3 (1–35)	20.9 ± 12.4 (4–70)

Males n (%)	2 (66.7)	5 (50)	20 (58.8)

***Clinical n (%)***			

Dystonia	3 (100)	10 (100)	33 (97)

*Site of onset*			

Cranial	0	2 (20)	9 (27.2)

Cervical	0	5 (50)	10 (30.3)

Limb	3 (100)	3 (30)	14 (42.4)

*Progression*			

Focal	0	1/10	0

Segmental	1 (33.3)	0	7 (21.2)

Generalized	2 (66.7)	9 (90)	26 (78.8)

Myoclonus	0	1 (10)	5 (14.7)

Choreoathetosis	0	0	1 (2.9)

Psychiatric disturbances	0	0	7 (20.6)

Intellectual disability	0	0	5 (14.7)

Epilepsy	0	0	1 (2.9)

Spasticity	0	0	1 (2.9)

Family history (n/%)	0	6 (60)	16 (47)

***MRI n (%)***			

Normal	2 (66.7)	10 (100)	26 (76.4)

GPi hypointensity	1 (33.3)	0	4 (11.7)

Cerebral atrophy	0	0	4 (11.7)

***Genetics n (%)***			

*Variants*	n = 3	n = 6	n = 20

Missense	0	4 (66.7)	0

Frameshift	1 (33.3)	2 (33.3)	10 (50)

Stop-gain	2 (66.7)	0	7 (35)

Splice-site loss	0	0	3 (15)

Homozygous	0	1 (16.7)	0

Heterozygous	3 (100)	5 (88.3)	20 (100)


^a^ mean ± standard deviation.

**Table 2:** Genetic profile of the cohorts.


	GENETIC VARIANT	PROTEIN LEVEL IDENTIFIER	

Chinese cohort	c.156 C > A	p.Asn52Lys	Missense

	c.692A>G	p.Tyr231Cys	Missense

	c.133_134dup	p.Pro46Alafs*6	Frameshift

	c.1929_1930del	p.Arg643fs*	Frameshift

	c.1661A > C	p.Lys554Thr	Missense

	c.2259C > G	p.Pro753Pro	Missense

European cohort	c.444_445del	p.Ala150Profs*11	Frameshift

	c.1394del	p.Leu465Argfs*89	Frameshift

	c.2181G > A	p.Trp727*	Stop-gain

	c.480delT	p.Asp161Thrfs*50	Frameshift

	c.1939C > T	p.Arg647*	Stop-gain

	c.1389C > G	p.Tyr463*	Stop-gain

	c.2140C > T	p.Gln714*	Stop-gain

	c.721_727del	p.Gly241Serfs*47	Frameshift

	c.1903C>T	p.Arg635*	Stop-gain

	c.244_259delinsGAGAGC	p.Lys82Glufs*124	Frameshift

	c.436del	p.Ile146Serfs*65	Frameshift

	c.455_462dup	p.Leu155Alafs*59	Frameshift

	c.559C > T	p.Arg187*	Stop-gain

	c.1094_1095dup	p.Tyr366Serfs*12	Frameshift

	c.1335 T > G	p.Tyr455*	Stop-gain

	c.1367 + 2 T > C		Splice-site loss

	c.1612–1G > C		Splice-site loss

	c.1720 + 1G > C		Splice-site loss

	c.1988_1989insG	p.Asn663Lysfs*2	Frameshift

	c.559C>T	p.Arg187*	Stop-gain

Indian cohort	c.600C>A, p.Arg525Ter c.2170_2171del	p.Tyr200* p.Arg525Ter p.Lys724Glufs*44	Stop-gain Stop-gain Frameshift


A review of two reported cases from India showed a median age of onset of 12.5 years (range- 10–15 years) and a median duration of 20 years. The predominant symptoms were dystonia that was crural in onset and got generalized at the time of presentation. There were no associated psychiatric symptoms, seizures, or myoclonus. There were no reported clinically apparent neuropsychological deficits, and any family history. The findings of formal neuropsychological testing were not available. One patient had response to levodopa. Brain MRI of one patient showed bilateral globus pallidus hypointensity and another patient had normal MRI. Genetic analysis in both patients showed stop-gain variants (c.600C>A;p.Tyr200* and p.Arg525Ter) in *VPS16* which were likely pathogenic.

### Chinese cohort (n = 10) [1,5,6,7]

There were 10 reported cases from China. The median age of presentation was 39 years (range- 9–65 years), the median age at onset was 10 years (range-8–60 years), the median duration of 13 years (1–35 years), and equal gender distribution. All 10 patients had dystonia as the initial symptom with cranial-onset in 2, cervical-onset in 5, and limb-onset in 3 patients. Nine patients progressed to generalized dystonia and 1 patient remained focal. One patient had a family history. Five affected patients were from one family. One patient had myoclonus along with dystonia. There were no associated psychiatric symptoms, or seizures. There were no reported clinically apparent neuropsychological deficits. The findings of formal neuropsychological testing were not available. Brain MRI was normal in all patients. There were 4 missense variants and 2 frameshift variants. One homozygous and 5 heterozygous variants were reported. There were 4 pathogenic variants, 1 variant of uncertain significance, and 1 benign variant.

### European cohort (n = 34) [8,9,10,11,12,13]

There were 34 reported cases from Europe. The median age of presentation was 45 years, the median age at onset was 14 years, the median duration of 26 years, and male predominance. Dystonia as the initial symptom was seen in all except one patient. Dystonia was cranial-onset in 9, cervical-onset in 10, and limb-onset in 14 patients. Twenty-six patients progressed to generalized dystonia and 7 patients progressed to segmental distribution. One patient had stereotypies as the initial symptom. Sixteen patients had a family history of dystonia. Five patients had myoclonus along with dystonia. Intellectual disability was seen in 5 patients and psychiatric symptoms in 7 patients. Nine patients had GPi deep brain stimulation (DBS), while 3 of them had good and 6 had partial response. The brain MRI were normal in 26 patients, cerebral atrophy in 4, and globus pallidus hypointensity in 4 patients. There were 10 frameshift variants, 7 stop-gain, and 3 splice-site loss. All variants were pathogenic and heterozygous.

## Discussion

The study aimed to describe the clinical, imaging, and genetic profile of patients with *VPS16*-associated dystonia from India and compare the findings with the Chinese and European cohorts. All three cohorts had similar ages at onset (10–14 years). The Indian and European cohorts had a slight male dominance, while the Chinese cohort had an equal sex distribution. The European cohort patients had a longer duration of symptoms than the Chinese and Indian cohorts (14 years vs 26 years). All three cohorts had dystonia as the initial symptom in all the patients. In all three cohorts, dystonic symptoms started in the limbs, followed by cervical onset in the Chinese and European cohorts. The majority of the patients progressed to generalized dystonia in all three cohorts (67–90%), and only 20.5–33.3% of patients remained segmental. Myoclonus was the next common movement disorder and was reported mainly from the European cohort. Intellectual disability was mainly reported from the European cohort. Psychiatric symptoms were reported in the European cohort (20.6%) but no cases were reported in the Indian or Chinese cohorts. GPi DBS was found to have partial to good response in the European cohort. The other genetic dystonic syndromes with proven benefit from DBS are DYT1, DYT6, DYT11, DYT25, DYT 28 [14].

Brain MRI is normal in the majority of the cases from all the 3 cohorts. The abnormalities that were observed were GPi mineralization and cerebral atrophy, primarily reported from the European cohort. Missense variants were reported in the Chinese cohort with 5 patients from a single affected family having homozygous missense variant (c.156 C > A (p.Asn52Lys) [9]. There were five patients with missense variants who had generalized dystonia, two of whom had segmental dystonia, and one had focal dystonia. Frameshift variants and stop-gain variants were common in the European and Indian cohorts. Segmental dystonia was seen in the frameshift variants (70%) more than the generalized dystonia. Stop-gain variants had generalized dystonia rather than remaining segmental. The majority of the variants identified in all three cohorts were heterozygous loss of function variants that exhibited autosomal dominant inheritance. The “homotypic fusion and protein sorting” (HOPS) complex is the tethering protein complex that mediates docking interactions among late endosomes, lysosomes, and autophagosomes in the cytoplasm. The HOPS complex is composed of four “vacuolar protein sorting (VPS) class C subunits” which are VPS11, VPS 16, VPS18, and VPS 33A and two additional subunits (i.e., VPS39 and VPS41) [15,16,17]. Variants in the genes encoding for the HOPS complex have been associated with the etiopathogenesis of inherited dystonia (i.e., VPS16, VPS41, and VPS11) [18,19,20].

On analysis of all 47 patients with *VPS16*-associated dystonia from 3 cohorts, the median age at onset was 14 years (range-3–60 years), the median duration of 21 years (range-1–70 years), and male predominance (57.4%). Dystonia was prevalent among all patients with limb-onset dystonic symptoms (42.5%), followed by cervical-onset (31.9%). Generalized dystonia was the common topographic distribution seen in 79% of patients. The associated symptoms were myoclonus, choreoathetosis, stereotypies, intellectual disability, seizure, and psychiatric symptoms. The heterozygous frameshift variant was the common variant followed by the stop-gain variant. There have been 29 variants reported so far. Haploinsufficiency is caused by monoallelic variants of *VPS16*, which manifests as a dominant inheritance with incomplete penetrance [11].

The strength of the study was the detailed clinical, radiological, and genetic description of cases from all the three cohorts. The limitations of the study were the small sample size of the Indian and Chinese cohorts, variability in the number of reported cases among the groups, and lack of long-term follow-up of the Indian cohort.

## Conclusion

In our study, there were few differences in the phenotypic presentation of VPS16-associated dystonia across geographical regions, but the European cohort had a higher prevalence of myoclonus, intellectual disability, and psychiatric symptoms. Segmental dystonia was common among patients with frameshift variants, while generalized dystonia was common among patients with stop-gain variants.

## Data Accessibility Statement

The data will be made available on request.
